# Tar

**Published:** 1895-03-09

**Authors:** 


### The Panacea in English Medicine.

### III.—Tar.

It has been already noticed that it is in the non-professional practitioner that faith in a universal medicine is most likely to be found. The regularly qualified physician, even though in practice he be detected relying almost exclusively on one drug, will be found to guard himself carefully from suspicion of a credulity commonly despised. Accordingly, it is in the works of a man of science and vast learning, a mathematician, a philosopher, and a bishop, but no student of medicine, that the most open confession of faith is to be found in the catholicity and unfailing action of a favourite remedy. To celebrating the virtues of Tar-water, Bishop Berkeley devoted not only one of his most famous philosophical treatises and much of his time and correspondence for a period of twelve years, but the health of himself, his family, his friends, and his diocese.

The rise and progress of this singular enthusiasm in a man so remote in temperament and mental training from charlatanism, finds no parallel in medical history. The attention of Berkeley was first called to the medical properties of tar during a sojourn among the Indians as a young man in the Narragansett country. Years later as Bishop of Cloyne, among a starving and diseased population of Irish, remote from medical aid, and unacquainted with drugs, he began to feel the urgent need of supplying some measure of relief to the suffering poor of his diocese. An epidemic sickness which he describes as a bloody flux, of the nature it may be supposed of a dysenteric disorder, had taken hold of the population, enfeebled by recent famine. The Bishop accordingly began to experiment, very cautiously at first, with his new remedy. A heaped spoonful of rosin, powdered fine, was administered in a little broth, and the results were so surprisingly satisfactory and chimed in so happily with certain philosophical reflections of the Bishop's leisure hours, that he speedily began to urge his remedy upon all afflicted with the same disorder. Certain modifications of the dose soon suggested themselves. Broth was found to be an unsatisfactory medium, and the rosin or tar was administered in oil, or honey, until the Bishop had satisfied himself that water was the proper menstruum, by which the vital element contained in tar might be drawn off and conveyed into the human system. His recipe for making his famous tar-water was simplicity itself. "Pour a gallon of cold water on a quart of tar, and stir and mix them thoroughly with a wooden ladle or flat stick for the space of five or six minutes, after which the vessel must stand close covered and unmoved three days and nights that the tar may have time to subside, and then the clear water, having been first carefully skimmed without shaking the vessel, is to be poured off and kept for use, no more being made from the same tar." Before long the demands made upon the Bishop for his pet drink were so numerous that an apparatus for manufacturing it was set up in one of the rooms of the palace at Cloyne. He now began to extend the field of his experiments. Remembering that the Indians believed in its efficacy for small-pox, he tried it on his neighbours during an outbreak of this disorder, and found it a successful "preventive or preparative." "Several," he says, "were preserved from taking the small-pox by the use of this liquor; others had it in the mildest manner; and others, that they might be able to take the infection, were obliged to intermit drinking the tar-water." It was to be taken night and morning, about half a pint, on an empty stomach.

But the Bishop could not stop here. "It seemed probable," he thought, "that it might be also useful in other foulnesses of the blood." He began to administer it accordingly "in a great variety of cases"; for cutaneous eruptions, for ulceration of the bowels, for a consumptive cough, for pleurisy and peripneumonia; for indigestion, asthma, hysteria, gravel, and dropsy. In short, he was now fairly launched into a course of practically unlimited faith in the remedy he was permitted to introduce to mankind. He acknowledges himself to be "most sincerely persuaded that tar-water may be drank with great safety and success for the cure or relief of most, if not all diseases." Only his caution as a scientific man withheld him from an even stronger confession of faith. "I do not *say* it is a panacea; I only suspect it to be so—time and trial will show."

To reasoning powers so acute as Berkeley's and so profoundly exercised in searching into metaphysical truths, observed effects were not sufficient. Side by side with his medical experiments grew up a chain of reasoning, resulting in the publication of "Siris," which, to quote a writer in "Notes and Queries," "announced as an Essay on Tar-water, begins with tar and ends with the Trinity, the *omne scibile* forming the interspace." In this remarkable work are involved "some of the most subtle botanical, chemical, physiological, optical, and mechanical speculations of its time," combined with the highest thoughts in metaphysics and theology. Tar is the text or groundwork upon which the whole structure is built. As the vital essence of the world, it is calculated, he claims, to yield a water of health suited to every constitution and inimical to all disease.

The celebrity of the treatise as a contribution to medical science was extraordinary. Numerous editions were called for, and it was at once translated into French, German, Dutch, and Portuguese. In England it became the sensation of the hour. High and low, rich and poor, weak and strong, were seized with the tar mania. Large factories were started for making it after the good Bishop's recipe, and the quantity imbibed by its votaries seems to have been truly prodigious. There was, indeed, no limit to its scope; those who were well drank it that they might remain so, and few of the sick doubted but that with a sufficiency of draughts they might be rid of their disease. Tar-water was, in fact (probably all unsuspected by Cowper), the original cup "to cheer but not inebriate" ("Siris," section 217; 1744), although the nauseousness of the draught might lead some to question its hilarious effects.

The opposition of the faculty, rising in the progress of the controversy to acrid animosity, had little effect in damping public ardour or convincing the Bishop against his will. The craze did not entirely fade away till long after the Bishop's death in 1753. His own wretched health in later life, and that of his family, "over-tarred," it may be suspected, in their youth, may have caused him some misgivings at the end as to his panacea. It is now chiefly remembered as having given birth to the most erudite, and the most beautiful of George Berkeley's works.

